# Case Study: Efficacy of Engineering Controls in Mitigating Diacetyl and 2,3-Pentanedione Emissions During Coffee Grinding

**DOI:** 10.3389/fpubh.2022.750289

**Published:** 2022-05-18

**Authors:** Marcia L. Stanton, Tia L. McClelland, Michael Beaty, Anand Ranpara, Stephen B. Martin

**Affiliations:** Respiratory Health Division, National Institute for Occupational Safety and Health, Morgantown, WV, United States

**Keywords:** engineering control, coffee, grinding, diacetyl, 2, 3-pentanedione

## Abstract

Exposure to elevated levels of diacetyl in flavoring and microwave popcorn production has been associated with respiratory impairment among workers including from a severe lung disease known as obliterative bronchiolitis. Laboratory studies demonstrate damage to the respiratory tract in rodents exposed to either diacetyl or the related alpha-diketone 2,3-pentanedione. Respiratory tract damage includes the development of obliterative bronchiolitis-like changes in the lungs of rats repeatedly inhaling either diacetyl or 2,3-pentanedione. In one flavored coffee processing facility, current workers who spent time in higher diacetyl and 2,3-pentanedione areas had lower lung function values, while five former flavoring room workers were diagnosed with obliterative bronchiolitis. In that and other coffee roasting and packaging facilities, grinding roasted coffee beans has been identified as contributing to elevated levels of diacetyl and 2,3-pentanedione. To reduce worker exposures, employers can take various actions to control exposures according to the hierarchy of controls. Because elimination or substitution is not applicable to coffee production facilities not using flavorings, use of engineering controls to control exposures at their source is especially important. This work demonstrates the use of temporary ventilated enclosures around grinding equipment in a single coffee roasting and packaging facility to mitigate diacetyl and 2,3-pentanedione emissions from grinding equipment to the main production space. Concentrations of diacetyl and 2,3-pentanedione were measured in various locations throughout the main production space as well as inside and outside of ventilated enclosures to evaluate the effect of the enclosures on exposures. Diacetyl and 2,3-pentanedione concentrations outside one grinder enclosure decreased by 95 and 92%, respectively, despite ground coffee production increasing by 12%, after the enclosure was installed. Outside a second enclosure, diacetyl and 2,3-pentanedione concentrations both decreased 84%, greater than the 33% decrease in ground coffee production after installation. Temporary ventilated enclosures used as engineering control measures in this study effectively reduced emissions of diacetyl and 2,3-pentanedione at the source in this facility. These findings motivated management to explore options with a grinding equipment manufacturer to permanently ventilate their grinders to reduce emissions of diacetyl and 2,3-pentanedione.

## Introduction

Identification of obliterative bronchiolitis among former workers of a coffee processing facility that roasted, ground, flavored, and packaged coffee (1) prompted a National Institute for Occupational Safety and Health (NIOSH) Health Hazard Evaluation (HHE) at the facility in 2012. Findings from this evaluation of a flavored coffee production facility demonstrated excess shortness of breath and obstruction on spirometry, and respiratory illness was associated with exposure to elevated levels of diacetyl and 2,3-pentanedione in the flavoring room as well as in other areas of the facility where unflavored coffee was produced (2–4). Dissemination of findings from this evaluation prompted the submission of HHE requests by both owners/management and employees from other coffee production facilities requesting assistance in characterizing potentially hazardous exposures. Between 2016 and 2018, NIOSH completed industrial hygiene and medical surveys at 17 such facilities. Worker exposures above the NIOSH recommended exposure limits (RELs) of 5.0 parts per billion (ppb) diacetyl and 9.3 ppb 2,3-pentanedione (5) were measured in coffee roasting and packaging facilities of varying sizes and production volumes during the NIOSH HHEs (6). Grinding roasted coffee beans was a primary activity resulting in elevated worker exposures to diacetyl (6). In addition to diacetyl and 2,3-pentanedione, emissions of other volatile organic compounds and gases such as carbon monoxide can occur during activities in coffee roasting facilities (3, 7–13). NIOSH researchers provided each facility with results from the comprehensive surveys including recommendations based on the hierarchy of controls. We recommended use of engineering controls to protect employees from exposures associated with grinding. However, certain factors such as various production volumes, sizes of the facilities and associated grinding equipment, facility layouts, and levels of automation made it challenging to recommend a “one-size-fits-all” control strategy.

Solutions for controlling exposures usually follow the principles of the hierarchy of controls. NIOSH researchers often recommend the use of engineering controls to protect workers especially in workplaces where it is not possible to physically remove (eliminate) the hazard or replace (substitute) the hazard with an alternative material that is not hazardous or less hazardous. The NIOSH Engineering Controls Program (https://www.cdc.gov/niosh/programs/eng/default.html) works with a variety of partners to reduce exposures by focusing on engineering control recommendations. This group promoted the use of engineering controls for diacetyl and other food flavorings to industry, regulatory agencies, and consensus standard bodies (14) and in 2015 published a best practices engineering control document (15). This NIOSH Best Practices document (15) included specific engineering control and work practice guidance focused on flavoring production industries. Many of the controls used in the coffee flavoring industry involve ventilation to remove the contaminant and introduce replacement air. Specific considerations included ensuring (1) areas where flavorings are used remain under negative pressure relative to rest of space, (2) air from mixing rooms is not recirculated and is exhausted outdoors, (3) use of ventilated enclosures to collect dusts and vapors, (4) correct positioning of local exhaust ventilation (LEV) hoods, and (5) monitoring of workers' exposures to assess effectiveness of the system. Many of the recommendations NIOSH made in their coffee facility HHE reports were consistent with those in the Best Practices guidance. NIOSH also recommended that facilities implement comprehensive respiratory protection programs in the event respirators were needed until effective engineering and administrative controls were in place to keep diacetyl and 2,3-pentanedione exposures below their respective RELs.

The work described herein demonstrates the utility of ventilated enclosures to reduce grinding emissions at one roasting and packaging facility working to implement workplace changes in response to recommendations made by NIOSH.

## Context

NIOSH researchers contacted facilities where earlier HHEs were conducted to assist with development or evaluation of engineering control solutions to reduce worker exposures to diacetyl and 2,3-pentanedione. We were particularly interested in helping companies implement NIOSH recommendations to further enable the knowledge generated during the HHEs to be transferred into practice that could be utilized throughout the industry. This case study describes work performed at one facility interested in controlling emissions released from coffee grinders. The roasting and packaging facility did not produce flavored coffee products, so all diacetyl and 2,3-pentanedione exposures were from naturally produced sources. NIOSH researchers installed temporary ventilated enclosures around two large coffee grinders in this facility to demonstrate the effect of the control strategy to company management. As described herein, large reductions in airborne diacetyl and 2,3-pentanedione concentrations were obtained in nearly all areas of the facility. The reductions in diacetyl and 2,3-pentanedione concentrations provided sufficient evidence for company management to explore options to either isolate or ventilate the coffee grinders permanently.

### Work Area

Production activities including roasting, grinding, and packaging took place in an open area ~48,000 square feet/4,459 square meters. The green bean storage area was separated from the main production space by a wall with openings on each end. Approximately 50,000 pounds (22,679 kg) of whole coffee beans were roasted per day and ~55,000 pounds (24,948 kg) were ground over the 3-day period. Coffee was ground using three industrial-scale coffee grinders, each capable of grinding 600–700 pounds (272–318 kg) of roasted coffee beans per hour.

### Sampling Approach

We divided the main production area into six work areas: green bean storage, roasting, grinding, packaging, product storage, and shipping. General area air samples were collected and analyzed for diacetyl and 2,3-pentanedione at 29 locations during each of the 3 consecutive days. Three samples were collected from green bean storage, 15 from roasting, 39 from packaging, three from product storage, three from shipping area, and 18 from grinding. Outdoor area samples were collected in two locations to ensure contaminated outside air was not being re-entrained into the workplace. Area sampling equipment was placed at breathing zone height at each location. According to modified OSHA Method 1013/1016, two glass silica-gel sorbent tubes were protected from light and connected in series to a sampling pump operated at a flow rate of 50 milliliters per minute (mL/min) with analysis by gas chromatography/mass spectrometry (16–18). Two consecutive 3-h samples were collected and a time-weighted average (TWA) concentration for the two combined samples was calculated. We assumed the results from the 6-h monitoring period reflected the average diacetyl and 2,3-pentanedione concentration across a full, 8-h work shift. Area samples collected on the 1st day served to establish baseline concentrations throughout the facility. Paired samples were collected at each of the three grinders to allow for sampling inside and outside enclosures. On the 1st day of sampling and for the third grinder, the paired grinding samples represented duplicate samples. After construction of the grinding enclosures for two of the grinders at the end of day one, the paired samples represented one sampler placed inside, and one outside of the enclosure.

### Enclosure Construction

**Figure 1:**
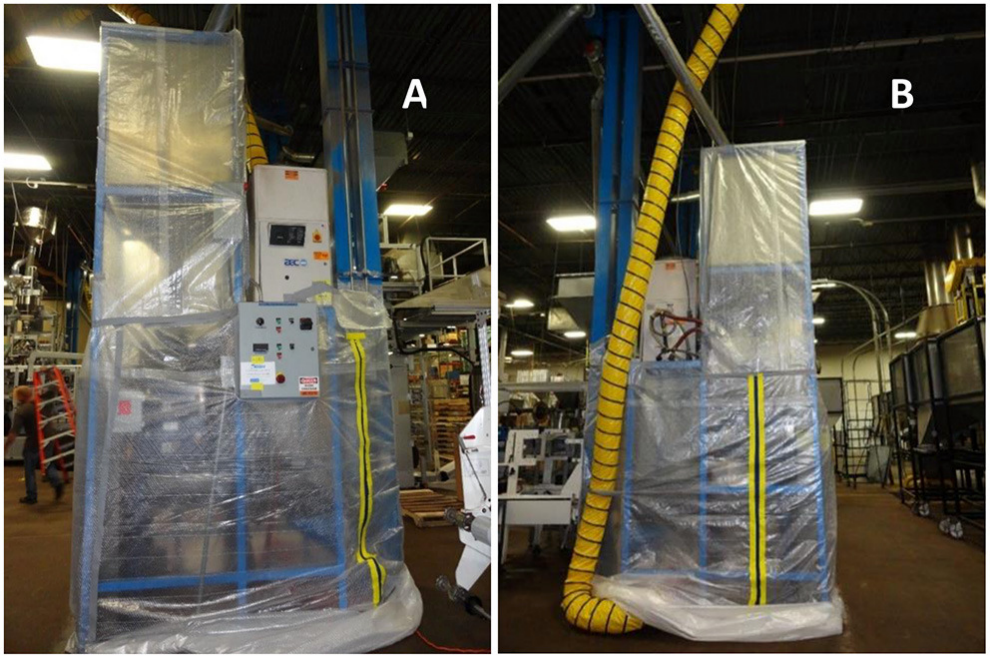
Images of Grinder B **(A)** front view and **(B)** side view on 2nd and 3rd day of sampling with enclosure in place. One sampler was located inside the enclosure and the second sampler was located immediately outside the enclosure. The yellow and black stripes are the zippers to allow employee access and the yellow flexible tube is a ventilation duct.

**Figure 2:**
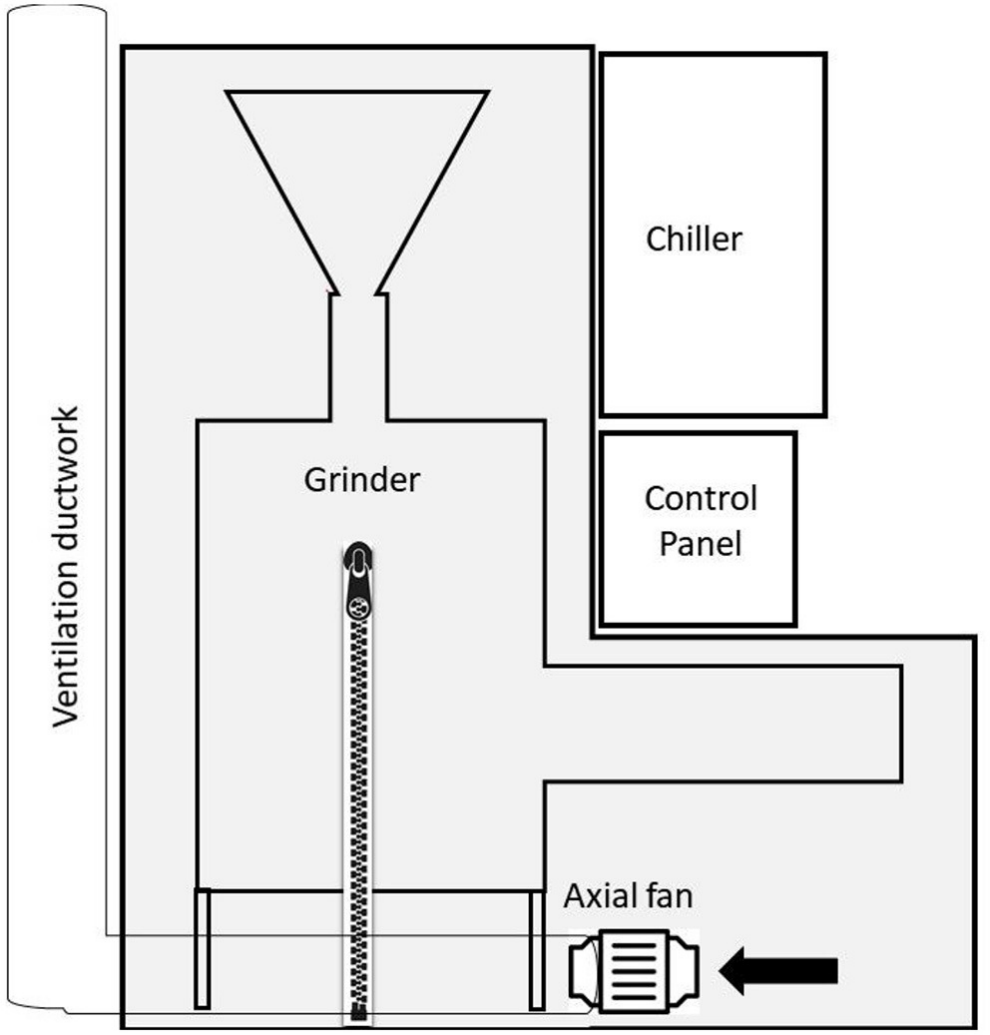
Schematic of ventilated enclosure design. The grinder equipment was enclosed depicted by area shaded in light gray. Both the chiller and control panel were located outside the enclosure. Each enclosure had two zippers to allow access. One axial fan per enclosure was placed on the floor under grinder equipment and connected to ventilation ductwork that was exhausted through the roof.

After completion of sampling on the 1st day, NIOSH investigators constructed temporary ventilated enclosures around two of the grinders (A and B) using reinforced plastic film and heavy-duty gaffer's duct tape (Figure 1). Each enclosure was fitted with two zippers to allow workers access to the grinder equipment to make necessary adjustments throughout the work shift. Exhaust ventilation from each enclosure was provided using an 8-inch (20-cm) diameter axial fan, typical of those used for confined space entry. One fan per enclosure was placed on the floor directly under the coffee grinders. Airflow inside the grinder A enclosure was 345 cubic feet per minute (cfm) [138 air changes per hour] and inside the grinder B enclosure was 330 cfm [126 air changes per hour]. Flexible ductwork was attached downstream of the fan that passed under the plastic enclosure and up to a large roof-top exhaust fan. This arrangement ensured the exhaust from inside the enclosure was released outside of the facility and not recirculated inside the space. Figure 2 shows a diagram of the grinder enclosure. No enclosure was constructed around the third grinder (C) because of logistical and space considerations. Grinder C was only operated briefly during sampling on the 2nd day and not operated on the 3rd day. All area samples on the 2nd and 3rd days were collected in the same locations as the 1st day.

### Data Analysis

We performed analyses using SAS version 9.4 (SAS Institute, Cary, NC), JMP 15.1.0 (SAS Institute Inc., Cary, NC), and Excel (Microsoft®, Redmond, WA). Diacetyl and 2,3-pentanedione concentrations for general area air samples are reported in parts per billion (ppb) by area location. Percent change concentrations at each grinder and by area location were calculated by subtracting the days 2 or 3 result concentration from day 1 and then dividing by the day 1 concentration.

## Results

### Diacetyl and 2,3-Pentanedione Concentrations by Work Area

**Figure 3:**
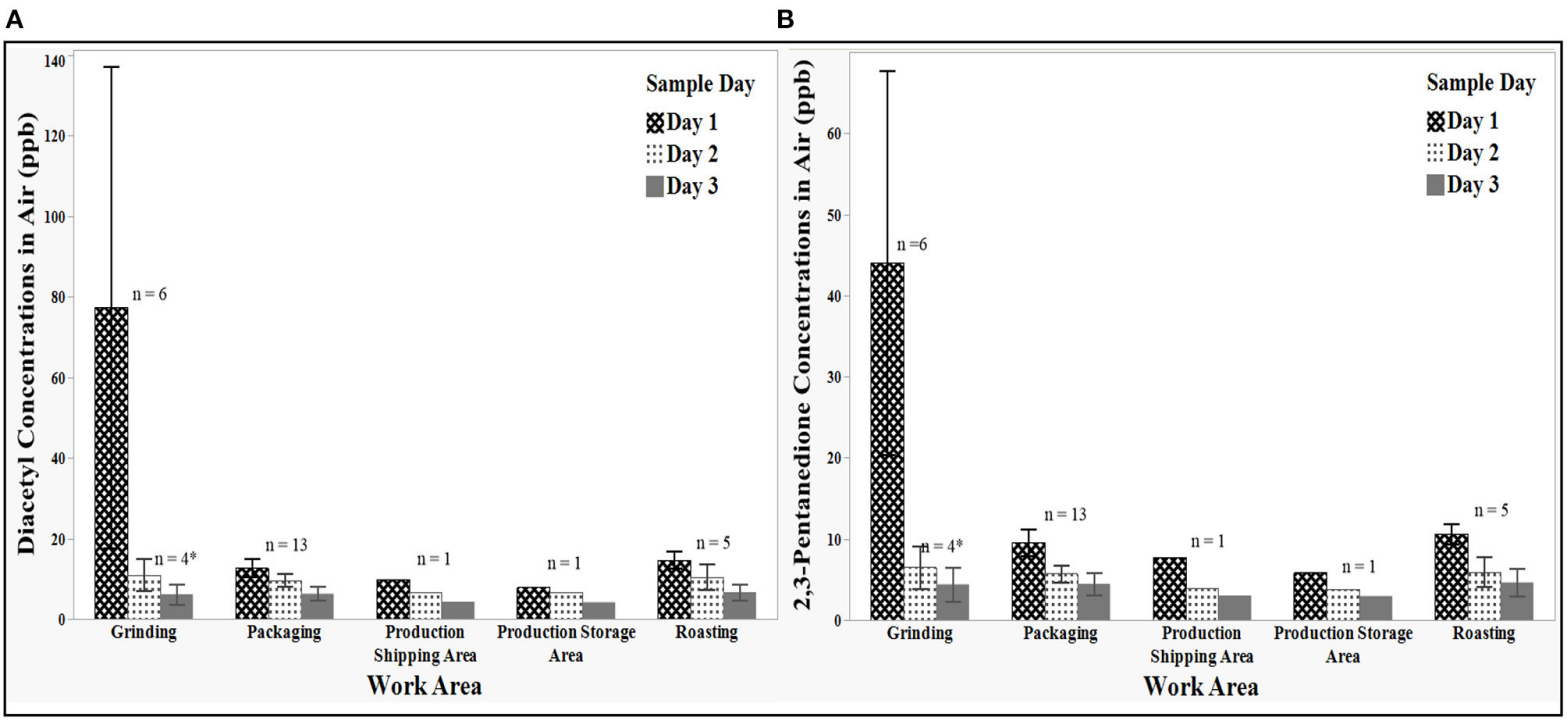
Mean concentrations of diacetyl **(A)** and 2,3-pentanedione **(B)** by sample day and work area. Error bars represent the standard deviation. *After enclosure, the grinding mean concentration on Days 2 and 3 did not include the sample inside the two grinding enclosures.

Twenty-nine area samples were collected on the 1st day prior to construction of the grinder enclosures to establish baseline concentrations throughout the main production area. Samples on the 2nd and 3rd day were collected in the same locations with ventilated enclosures around two of the three large grinders. Diacetyl and 2,3-pentanedione concentrations by day are shown in Figure 3.

On the 1st day of sampling, mean diacetyl concentrations were the highest in the grinding area at 77.4 ppb (range: 32.5–171.3 ppb). Compared to the grinding area, mean diacetyl concentrations were much lower in the other areas such as in roasting at 14.7 ppb (range: 12.7–17.7 ppb) and packaging at 12.7 ppb (range: 9.6–17.2 ppb). The mean diacetyl concentration in the production shipping area was 9.9 ppb and in the production storage area was 7.9 ppb.

Concentrations of 2,3-pentanedione followed a pattern similar to that of diacetyl on the 1st day of sampling. The highest 2,3-pentanedione concentrations were in grinding at 44 ppb (range: 26.6–87.1 ppb). In the roasting area, the mean 2,3-pentanedione concentration was 10.6 ppb (range: 9.1–12.3 ppb) and in packaging area the mean concentration was 9.6 ppb (range: 7.3–12. 5 ppb). The mean 2,3-pentanedione concentration in the production shipping area was 7.8 ppb and in the production storage area was 5.9 ppb.

On the 2nd day of sampling, diacetyl concentrations decreased in all sampling areas except in green bean storage area where there was a slight increase from 0.9 ppb on the 1st day to 1.0 ppb. The highest diacetyl concentrations were reported in grinding area at 10.9 ppb (range: 6.5–15.3 ppb) and roasting area at 10.5 ppb (range: 6.5–14.0 ppb). The third highest concentration was in packaging at 9.6 ppb (range: 7.1–14.1 ppb). The mean concentration in the production storage area was 6.7 ppb and in the production shipping area was 6.6 ppb.

Concentrations of 2,3-pentanedione on the 2nd day of sampling also decreased in all sampling areas except in green bean storage. The highest 2,3-pentanedione concentration was in grinding at 6.5 ppb (range: 3.9–10.0 ppb). The mean 2,3-pentanedione concentrations in roasting and packaging were similar at 6.0 ppb (range: 3.7–8.2 ppb) and 5.7 ppb (range: 4.3–8.7 ppb), respectively. The 2,3-pentanedione concentration was 4.0 ppb in the production shipping area and 3.8 ppb in the production storage area.

Concentrations of both diacetyl and 2,3-pentanedione continued to decrease on the 3rd day of sampling. Unlike the 1st and 2nd days of sampling, the highest mean diacetyl concentration on the 3rd day was in roasting at 6.6 ppb (range: 4.0–8.8 ppb). Diacetyl concentrations were slightly higher in packaging at 6.3 ppb (range: 2.8–9.0 ppb) than in grinding at 6.1 ppb (range: 3.0–8.3 ppb). The concentration of diacetyl was 4.3 ppb in the production shipping area and 4.2 ppb in the production storage area.

Concentrations of 2,3-pentanedione on the 3rd day trended with diacetyl. Unlike the 1st and 2nd days of sampling, the highest mean 2,3-pentanedione concentration on the 3rd day was in roasting at 4.6 ppb (range: 2.5–6.5 ppb), followed by packaging at 4.5 ppb (range: 1.7–6.2 ppb) then grinding at 4.4 ppb (range: 2.0–6.6 ppb). The concentration of 2,3-pentanedione was 3.0 ppb in both the production shipping area and in the production storage area.

Concentrations of diacetyl and 2,3-pentanedione were lowest in the green bean storage area ranging from 0.9 to 1.0 ppb and < 0.3 to 0.5 ppb, respectively. These results were not shown in Figure 3.

### Concentrations Inside and Outside of Temporary Grinder Enclosures

Concentrations of diacetyl and 2,3-pentanedione inside and outside of the temporary grinder enclosures are shown in Table 1. On the 2nd day of sampling, the concentration of diacetyl inside the grinder A enclosure was 721.2 ppb and the concentration immediately outside the enclosure was 15.3 ppb. The concentration of 2,3-pentanedione inside the grinder A enclosure was 335.1 ppb and the concentration immediately outside the enclosure was 10.0 ppb. At grinder B, the diacetyl concentration inside the enclosure was 907.2 ppb and the concentration immediately outside the enclosure was 12.9 ppb. The 2,3-pentanedione concentration inside the grinder B enclosure was 429.6 ppb and directly outside of the enclosure was 7.0 ppb. At grinder C (no enclosure), diacetyl concentrations from the two side-by-side samples were 6.5 and 8.7 ppb and 2,3-pentanedione concentrations were 3.9 and 5.2 ppb.

**Table 1:** Diacetyl and 2,3-pentanedione concentrations in parts per billion (ppb) inside and outside Grinder A & B enclosures.

**Location**	**Diacetyl concentration (ppb)**			**2,3-pentanedione concentration (ppb)**		
	Day 1^*^	**Day 2**	**Day 3**	Day 1^*^	**Day 2**	**Day 3**
	**No enclosure**			**No enclosure**		
Inside Grinder A enclosure	134.3	721.2	590.2	56.1	335.1	441.2
Outside Grinder A enclosure	171.3	15.3	8.3	87.1	10.0	6.6
Inside Grinder B enclosure	38.1	907.2	418.8	26.6	429.6	303.1
Outside Grinder B Enclosure	51.3	12.9	8.0	35.2	7.0	5.5

*One sample collected per day at each location (n = 1 per cell)*.

* *As there was no enclosure on day 1, the inside and outside samples represent duplicate samples. Samples were placed in the same locations once the enclosures were introduced*.

On the third day of sampling, the diacetyl concentration was 590.2 ppb and 2,3-pentanedione was 441.2 ppb inside the grinder A enclosure. Immediately outside the grinder A enclosure diacetyl was 8.3 ppb and 2,3-pentanedione was 6.6 ppb. At grinder B, diacetyl inside the enclosure measured 418.8 ppb and 2,3-pentanedione measured 303.1 ppb with 8.0 ppb diacetyl and 5.5 ppb 2,3-pentanedione immediately outside. At grinder C, the diacetyl concentration was 3.0 and 5.2 ppb and 2,3-pentanedione concentration was 2.0 and 3.5 ppb at the two side-by-side samplers on the 3rd day of sampling.

### Production Volumes and Percent Change in Diacetyl and 2,3-Pentanedione Concentrations

Total pounds of coffee roasted on the 2nd and 3rd days of sampling were comparable (within 4% compared to day 1). The amount of coffee packaged per day varied by only 8% across the 3 days. The total amount of coffee ground each day showed more variability, largely because of the limited use of grinder C. In total, 12% more coffee was ground on the 2nd day but 67% less on the 3rd day, compared to the 1st day. Compared to the 1st day, grinder A ground 58% more coffee on the 2nd day and 12% more on the 3rd day. The percent changes in diacetyl and 2,3-pentanedione concentrations and production volumes are shown in Table 2. Compared to day one, the diacetyl concentration measured just outside the grinder A enclosure showed a 91% decrease on the 2nd day and a 95% decrease on the 3rd day. Concentrations of 2,3-pentanedione decreased by 89% on the 2nd day and by 92% on the 3rd day. Compared to the 1st day, grinder B ground 349% more coffee on the 2nd day and 33% less coffee on the 3rd day. Diacetyl concentrations just outside the grinder B enclosure were reduced by 75% on the 2nd day and by 84% on the 3rd day. Concentrations of 2,3-pentanedione decreased by 80% on the 2nd day and by 84% on the 3rd day. Grinder C ground 73% less coffee on the 2nd day and no coffee at all on the 3rd day, with decreases in diacetyl concentrations of 78 and 88% and 2,3-pentanedione concentrations of 84 and 91% on the 2 days, respectively.

**Table 2:** Percent change in diacetyl and 2,3-pentanedione concentrations and production volumes across sampling days.

**Location**	**% Change**		**% Change**	
	**Days 1 to 2**		**Days 1 to 3**	
	**Diacetyl/2,3-pentanedione**	**Production volume**	**Diacetyl/2,3-pentanedione**	**Production volume**
Outside Grinder A enclosure	−91/−89	+58	−95/−92	+12
Outside Grinder B enclosure	−75/−80	+349	−84/−84	−33
Grinder C (no enclosure)^*^	−78/−84	−73	−88/−91	−100

* *Paired sample results were averaged in calculation*.

### Impact of Enclosure in Other Areas

**Figure 4:**
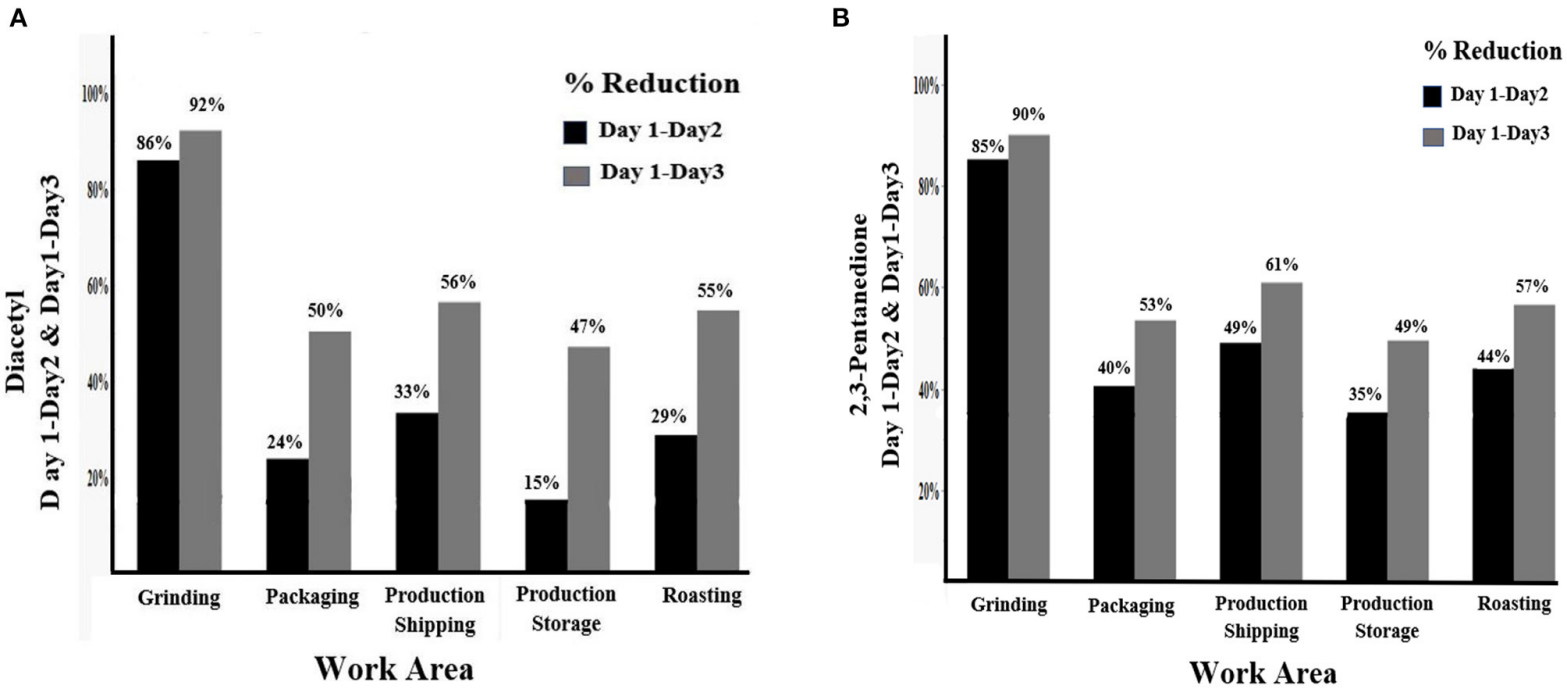
Percent reduction in mean concentrations of **(A)** diacetyl and **(B)** 2,3-pentanedione by work area between Days 1 and 2 and Days 1 and 3.

Overall concentrations of diacetyl and 2,3-pentanedione decreased in all sampled areas within the production space after the temporary ventilated enclosures were constructed (Figure 4). Diacetyl concentration reductions ranged from 15% in production storage to 33% in production shipping. For 2,3-pentanedione, concentration reduction ranged from 35% in production storage to 49% in the production shipping area.

Both diacetyl and 2,3-pentanedione concentrations had higher reductions from days 1 to 3 than from days 1 to 2. Diacetyl concentration reductions ranged from 47% in production storage to 56% in production shipping. 2,3-Pentanedione concentration reductions ranged from 49% in production storage to 61% in the production shipping area.

## Discussion

Grinding is a prominent activity at many coffee roasting and packaging facilities. NIOSH HHE investigations identified the activity of grinding as one of the main sources of emissions for alpha-diketones such as diacetyl and 2,3-pentanedione from both flavored and unflavored coffee that could contribute to worker exposures (6). Controlling exposures typically follows the five-step hierarchy of controls: elimination, substitution, engineering controls, administrative controls, and personal protective equipment. For coffee roasting and packaging facilities, elimination and substitution are typically not feasible because diacetyl and 2,3-pentanedione occur naturally in coffee and are generated during activities such as grinding and roasting. However, elimination and substitution of exogenous flavorings is a possible approach to limiting diacetyl and 2,3-pentanedione exposure related to the addition of flavorings. For this case study, we explored the use of engineering controls including enclosure and LEV at grinding machines as a method to reduce exposure in unflavored coffee production. Sampling results from the 1st day showed diacetyl concentrations at grinders were 5–10 times higher and 2,3-pentanedione concentrations were 4–7 times higher than in other areas of the production facility. Temporary enclosures constructed on two of three large grinders at this facility demonstrated that isolating grinders from the surrounding production space and exhausting that air directly outside can result in meaningful reductions in diacetyl and 2,3-pentanedione concentrations in air throughout the workplace and not just close to the enclosed grinders. The temporary enclosures used in this case study were constructed using plastic sheeting and duct tape, and exhaust ventilation was not optimized. Permanent, well-designed grinder enclosures with appropriate ventilation systems would likely result in more pronounced reductions in airborne alpha-diketone concentrations. To that end and based on the results of this evaluation, management at the facility communicated a desire to incorporate grinder enclosures aimed at reducing worker exposures.

Like previous studies utilizing ventilation control measures, this study measured substantial reductions in inhalational exposures to diacetyl and 2,3-pentanedione concentrations after installing ventilated enclosures. Fransman et al. created a database called the Exposure Control Efficacy Library (ECEL) that included 433 records from 90 peer-reviewed publications to examine efficacy values for six measures (i.e., enclosure, LEV, specialized ventilation, general ventilation, suppression, and worker separation) (19). In their analyses, enclosure and general ventilation had the lowest efficacies at 50 and 43%, respectively, while specialized ventilation and LEV had greater estimated efficacies at 87 and 82%. In the enclosure demonstration presented here, we measured reductions in diacetyl of 92% at the grinders and 79% in the overall production area and for 2,3-pentanedione 90% at the grinders and 77% in the overall production area.

Reductions in airborne diacetyl and 2,3-pentanedione concentrations throughout the plant were substantial after the grinders were enclosed although several factors were not controlled that could make the enclosure performance better than demonstrated here. As discussed, enclosures were constructed using simple plastic sheeting and duct tape. Although these materials permitted for easy and relatively quick construction, they did not allow for a completely sealed enclosure. Permanent enclosures should be specially designed according to grinder size, shape, and location in the production space, and employee access needs. The ventilation from the temporary enclosures was not optimized. The exhaust from each enclosure was simply the amount of air each fan could move with the ductwork attached and extended to the ceiling exhaust fan. This ventilation scheme was able to keep the enclosures under substantial negative pressure when the zippers were closed. However, it allowed substantial concentrations of diacetyl and 2,3-pentanedione to build up inside the enclosures that could put grinding personnel at substantial risk for exposure upon entry. Permanent enclosures could be designed with the exhaust flow necessary to maintain lower airborne concentrations. It is not clear whether those concentrations could be maintained low enough that workers would not have to wear respiratory protection when inside the enclosure.

This case study aligns with the NIOSH mission of preventing occupational illness by reducing exposures through controlling hazards in the workplace following the Prevention through Design Initiative (20). We were able to engage with company management to explore options for controlling an exposure and demonstrate the utility of process enclosure. Lessons learned during this exercise can be built upon to develop more permanent solutions designed specifically to control emissions in this industry.

### Limitations

The temporary enclosures were difficult for employees to enter, and interior space was limited making it hard to maneuver inside to make grinder adjustments. To gain access to the grinders, employees had to unzip the access zipper from the floor until the opening was large enough to enter; ultimately releasing high levels of diacetyl and 2,3-pentanedione into the larger production space, which likely resulted in higher concentrations in these compounds for other area samples taken in the plant. The number of times or length of time the enclosures were opened was not recorded during our sampling so we do not know the impact opening the enclosures may have had on other areas. Grinder operators were observed wearing air purifying half-face respirators fitted with organic vapor cartridges. Although we did not measure personal exposures, depending on concentrations within the enclosure and the amount of time an employee accessed the enclosure, a half-face respirator may not have been sufficient to reduce a 15-min time-weighted average exposure to below the short-term exposure limits for diacetyl or 2,3-pentanedione. The grinders were located on one side of the packaging areas but were not located directly beside each other. Each grinder was slightly different in their input and outlet points. The overall contaminant concentrations throughout the facility likely varied depending on which grinders were operating and for how long. Not being able to construct an enclosure around grinder C limited the ability to assess the full impact of having all the grinders enclosed. However, not having the third enclosure more than likely had a limited effect on results because grinder C was operated sparingly on the 2nd day and did not operate on the last day of sampling. Another limiting variable during this study was not having control of most coffee processing and packaging activities that occurred in the plant during sampling; daily production activities may have increased or decreased the air concentrations measured in this study. Concentrations of diacetyl and 2,3-pentanedione within coffee roasting and packaging facilities are subject to production levels and can vary daily or by time of year such as during holidays when production levels may be greater. Sampling for this scenario was only done for 3 days, which provided a limited number of samples. The results of this study are specific to this worksite and subject to the operating conditions during the 3 days of sampling.

## Conclusion

To our knowledge, this study was the first attempt to demonstrate the impact of using ventilated enclosures to remove grinder emissions in a coffee roasting and packaging facility. This project clearly showed that controlling airborne concentrations of diacetyl and 2,3-pentanedione released during coffee grinding substantially reduced emissions into the workplace. Controlling hazardous emissions at the source using ventilated enclosures was an effective means of reducing alpha-diketone emissions into the facility where workers could be exposed. These results motivated management to explore options with a grinding equipment manufacturer to permanently ventilate their grinders to reduce emissions of diacetyl and 2,3-pentanedione. This work highlights the utility of a research-to-practice intervention that could be considered at other coffee roasting and processing facilities interested in controlling emissions during coffee grinding.

## Data Availability Statement

The datasets presented in this article are not readily available because, due to restrictions imposed under the US privacy act and the limitations of what participants consented to, the data underlying the analyses presented, beyond what is provided in the paper, are confidential and not available to researchers outside the National Institute for Occupational Safety and Health (NIOSH). For more information about NIOSH's policy regarding sensitive data, see https://www.cdc.gov/niosh/ocas/datahandle.html. Requests to access the datasets should be directed to MS, zfc5@cdc.gov.

## Author Contributions

MS, TM, MB, and SM designed the study, collected the samples, analyzed the data, and prepared the manuscript. AR analyzed the samples and assisted with preparation of manuscript. All authors approved the article for publication.

## Funding

This work was supported by the National Institute for Occupational Safety and Health (NIOSH).

## Author Disclaimer

The findings and conclusions in this report are those of the authors and do not necessarily represent the views of the National Institute for Occupational Safety and Health (NIOSH). Mention of any company or product does not constitute endorsement by NIOSH. In addition, citations to Web sites external to NIOSH do not constitute NIOSH endorsement of the sponsoring organizations or their programs or products. Furthermore, NIOSH is not responsible for the content of these Web sites.

## Conflict of Interest

The authors declare that the research was conducted in the absence of any commercial or financial relationships that could be construed as a potential conflict of interest.

## Publisher's Note

All claims expressed in this article are solely those of the authors and do not necessarily represent those of their affiliated organizations, or those of the publisher, the editors and the reviewers. Any product that may be evaluated in this article, or claim that may be made by its manufacturer, is not guaranteed or endorsed by the publisher.

## References

[1] CDC. Obliterative bronchiolitis in workers in a coffee-processing facility - Texas, 2008-2012. MMWR Morb Mortal Wkly Rep. (2013) 62:305–7. 23615673 PMC4604960

[2] Bailey R Cox-Ganser J Duling M Lebouf R Martin S Green B . Respiratory morbidity in a coffee processing workplace with sentinel obliterative bronchiolitis cases. Am J Indus Med. (2015) 58:1235–45. 10.1002/ajim.22533 26523478 PMC4715657

[3] Duling MG Lebouf RF Cox-Ganser JM Kreiss K Martin SB Bailey RL. Environmental characterization of a coffee processing workplace with obliterative bronchiolitis in former workers. J Occup Environ Hyg. (2016) 13:770–81. 10.1080/15459624.2016.1177649 27105025 PMC5836548

[4] NIOSH. Evaluation of Exposures and Respiratory Health at a Coffee Roasting, Flavoring, and Packaging Facility. Morgantown, WV: U.S. Department of Health and Human Services, Centers for Disease Control and Prevention, National Institute for Occupational Safety and Health (2020).

[5] NIOSH. Criteria for a Recommended Standard: Occupational Exposure to Diacetyl and 2,3-Pentanedione. Cincinnati, OH (2016).

[6] Lebouf RF Blackley BH Fortner AR Stanton M Martin SB Groth CP . Exposures and emissions in coffee roasting facilities and cafes: diacetyl, 2,3-pentanedione, and other volatile organic compounds. Front Public Health. (2020) 8:561740. 10.3389/fpubh.2020.561740 33072698 PMC7531227

[7] Newton J. Carbon monoxide exposure from coffee roasting. Appl Occup Environ Hyg. (2002) 17:600–2. 10.1080/10473220290095899 12216587

[8] Akiyama M Murakami K Ohtani N Iwatsuki K Sotoyama K Wada A . Analysis of volatile compounds released during the grinding of roasted coffee beans using solid-phase microextraction. J Agric Food Chem. (2003) 51:1961–9. 10.1021/jf020724p 12643659

[9] Nishimura F Abe S Fukunaga T. Carbon monoxide poisoning from industrial coffee extraction. J Am Med Assoc. (2003) 290:334–334. 10.1001/jama.290.3.334 12865373

[10] Daglia M Papetti A Aceti C Sordelli B Spini V Gazzani G. Isolation and determination of alpha-dicarbonyl compounds by RP-HPLC-DAD in green and roasted coffee. J Agric Food Chem. (2007) 55:8877–82. 10.1021/jf071917l 17927199

[11] Raffel J Thompson J. Carbon monoxide from domestic coffee roasting: a case report. Ann Intern Med. (2013) 159:795–6. 10.7326/0003-4819-159-11-201312030-00023 24297206

[12] Hawley B Cox-Ganser JM Cummings KJ. Carbon monoxide exposure in workplaces, including coffee processing facilities. Am J Respir Crit Care Med. (2017) 196:1080–1. 10.1164/rccm.201703-0513LE 28471692 PMC5649989

[13] Lebouf RF Aldridge M. Carbon monoxide emission rates from roasted whole bean and ground coffee. J Air Waste Manag Assoc. (2019) 69:89–96. 10.1080/10962247.2018.1515125 30148693 PMC6430709

[14] NIOSH. Engineering Controls Program. Cincinnati, OH: U.S. Department of Health and Human Services, Centers for Disease Control and Prevention, National Institute for Occupational Safety and Health (2016).

[15] NIOSH. Best practices: engineering controls, work practices and exposure monitoring for occupational exposures to diacetyl and 2,3- pentanedione. Cincinnati, OH: P.H.S. U.S. Department of Health and Human Services, Centers for Disease Control and Prevention, National Institute for Occupational Safety and Health (2015).

[16] OSHA. Method 1013: Acetoin and Diacetyl. (2008). Available online at: https://www.osha.gov/dts/sltc/methods/validated/1013/1013.pdf (accessed April 23, 2018).

[17] OSHA. Method 1016: 2,3-Pentanedione. (2010). Available online at: https://www.osha.gov/dts/sltc/methods/validated/1016/1016.pdf (accessed April 23, 2018).

[18] Lebouf RF Simmons M. Increased sensitivity of OSHA method analysis of diacetyl and 2,3-pentanedione in air. J Occup Environ Hyg. (2017) 14:343–8. 10.1080/15459624.2016.1252846 27792470 PMC5778437

[19] Fransman W Schinkel J Meijster T Van Hemmen J Tielemans E Goede H. Development and evaluation of an exposure control efficacy library (ECEL). Ann Occup Hyg. (2008) 52:567–75. 10.1093/annhyg/men054 18703542

[20] Schulte PA Rinehart R Okun A Geraci CL Heidel DS. National Prevention through Design (PtD) Initiative. J Safety Res. (2008) 39:115–21. 10.1016/j.jsr.2008.02.021 18454950

